# Siderophore biosynthesis coordinately modulated the virulence-associated interactive metabolome of uropathogenic *Escherichia coli* and human urine

**DOI:** 10.1038/srep24099

**Published:** 2016-04-14

**Authors:** Qiao Su, Tianbing Guan, Haitao Lv

**Affiliations:** 1 The Laboratory for Functional Omics and Innovative Chinese Medicine, Innovative Drug Research Center, Chongqing University, Chongqing 401331, P.R. China; 2 Shanghai Center for Systems Biomedicine, Key Laboratory of Systems Biomedicine (Ministry of Education), Shanghai Jiao Tong University, Shanghai 200240, China

## Abstract

Uropathogenic *Escherichia coli* (UPEC) growth in women’s bladders during urinary tract infection (UTI) incurs substantial chemical exchange, termed the “interactive metabolome”, which primarily accounts for the metabolic costs (utilized metabolome) and metabolic donations (excreted metabolome) between UPEC and human urine. Here, we attempted to identify the individualized interactive metabolome between UPEC and human urine. We were able to distinguish UPEC from non-UPEC by employing a combination of metabolomics and genetics. Our results revealed that the interactive metabolome between UPEC and human urine was markedly different from that between non-UPEC and human urine, and that UPEC triggered much stronger perturbations in the interactive metabolome in human urine. Furthermore, siderophore biosynthesis coordinately modulated the individualized interactive metabolome, which we found to be a critical component of UPEC virulence. The individualized virulence-associated interactive metabolome contained 31 different metabolites and 17 central metabolic pathways that were annotated to host these different metabolites, including energetic metabolism, amino acid metabolism, and gut microbe metabolism. Changes in the activities of these pathways mechanistically pinpointed the virulent capability of siderophore biosynthesis. Together, our findings provide novel insights into UPEC virulence, and we propose that siderophores are potential targets for further discovery of drugs to treat UPEC-induced UTI.

A urinary tract infection (UTI), one of the most prevalent infectious diseases, adversely affects the health of 60–70% of women worldwide^1^,^2^,^3^. Uropathogenic *Escherichia coli* (UPEC) accounts for approximately 70–80% of clinical cases^4^,^5^,^6^. Drug resistance has developed to more than 70% of the antibiotics used against UPEC-induced UTI, which is difficult to treat because of its unclear pathogenesis and low-efficiency drugs^7^. Because the essential medium for UPEC growth is the urine in women’s bladders^8^,^9^, human urine is thought to have a strong biological effect on the growth of UPEC, as it has been observed that the composition of human urine was one of key determinants of the antibacterial activity of siderocalin *in vivo*, and it has also been shown to regulate the expression and functions of the type 1 pili by which UPEC is capable of colonizing the bladder during infection^10^,^11^. Generally, UPEC growth in human urine incurs substantial chemical exchange, termed the “interactive metabolome”, which primarily accounts for the metabolic costs (utilized metabolome)^6^ and metabolic donations (excreted metabolome)^3^ between them. The interactive metabolome might have a role in UPEC virulence because human urine provides the essential carbon source for UPEC growth *in vivo*. However, it is still poorly understood whether the interactive metabolome that has been implicated in UTIs caused by UPEC is markedly different from those of non-UPEC and human urine. In the present study, we attempted to determine whether the individualized interactive metabolome between UPEC and human urine could be significantly distinguished from that of non-UPEC to provide a better understanding of UPEC virulence and associated UTI. In addition, siderophores are chemically diverse secondary metabolites that are broadly biosynthesized by a variety of pathogenic microbes, plants and fungi; their primary functions are to assist the biological organisms in capturing iron to maintain their normal growth and development^12^,^13^,^14^,^15^,^16^,^17^. In recent years, there has been increasing evidence that chemical diversity determines the functional variety of siderophores, in addition to chelating iron. These novel functions are involved in regulating system metabolism, molecular signalling, and pathogen virulence^6^,^16^,^18^,^19^. In pathogenic microorganisms, siderophores have been clearly confirmed as promoters of host-pathogen interactions, as new effectors enforced the virulence of pathogenic strains against the host^20^,^21^,^22^,^23^,^24^,^25^. UPEC was reported to account for approximately 70–80% of clinical cases of UTI, and most of the UPEC strains produced some or all of the following four siderophores: aerobactin, yersiniabactin, salmochelin and enterobactin^4^,^6^,^20^,^26^. They are abundantly produced by many pathogenic strains as well^27^,^28^,^29^, and our previous studies revealed that these siderophores were significantly associated with UPEC virulence by systematically modulating central metabolism^4^,^5^,^6^. Importantly, our data suggested that yersiniabactin and salmochelin have the capacity to be developed as novel targets for virulence-based treatments and antibiotics against UTI^4^,^5^,^6^, and in this study we also aimed to determine whether those siderophores participate directly in modulating the virulence-associated interactive metabolome between UPEC and human urine. The schematic illustration for the strategy used in this study is shown in Fig. 1.

## Materials and Methods

### Chemicals and reagents

High-purity D_2_O was purchased from Cambridge Isotope Laboratories (Cambridge, USA). Methanol (HPLC-grade) and acetonitrile (HPLC-grade) were purchased from Fisher Scientific (Loughborough, UK). LB broth and LB agar were purchased from Difco Laboratories (Franklin Lakes, USA).

### Human urine collection and preparation

Urine samples were donated by healthy adult volunteers (Table S1). This study was approved by the Institutional Review Board of Chongqing University and the Human Subjects Review Committee of Qianjiang Center for Disease Control & Prevention. The volunteers signed written informed consent for collection of one morning specimen. The strictly defined collection criteria excluded those who were undergoing antibiotic therapy, were pregnant, had recently suffered from a UTI, or had kidney disease. The urine samples were pooled together for the first experiment. The pooled urine was centrifuged and consecutively sterilized with 0.45-μm and 0.22-μm filters; the sterilized urine was used as the nutritional medium for the strains’ culture. All experiments were performed in accordance with the guidelines for human-subject-based biological experiments issued by the Institutional Review Board of Chongqing University and the Human Subjects Review Committee of Qianjiang Center for Disease Control & Prevention.

### Bacterial strains and human urinary culture

Table S2 lists the UPEC and associated mutant strains used in this study. The experimental samples were collected according to procedures from our previous studies^5^,^6^, with some modifications. Briefly, all the strains were statically incubated on LB agar plates for 16 h, and then one colony was removed from each plate and transferred into 4 ml of LB broth and incubated for 4 h with shaking at 37 °C. Subsequently, 50 μl of the culture was added to 50 ml of pooled urine and incubated for 18 h with shaking at 37 °C. Finally, all the samples were centrifuged to isolate the supernatant for the NMR-based metabolomic assay. The Institutional Review Board of Chongqing University approved this study. All experiments were performed in accordance with the guidelines for biological experiments issued by The Institutional Review Board of Chongqing University.

### Construction of the UPEC mutant strains

The iron acquisition genes were deleted from *E. coli* 83972 using the previously described λ Red recombinase gene inactivation method^20^. The pKD4 plasmid was used as the template for the siderophore biosynthetic gene deletion mutants (the primers are listed in Table S3)^5^,^30^. The pCP20 plasmid was used to remove the antibiotic resistance cassette. The single, double, triple and quadruple siderophore mutants were constructed as described in a previous study^20^. All deletion mutants were verified by PCR and DNA sequencing as described in a previous study^20^.

### Sample preparation for the NMR-based metabolomics assay

Pooled control urine samples (pre-culture) and 2 ml of the supernatants isolated from all the culture samples were lyophilized completely and then mixed with 600 μl of 100% D_2_O-PBS solution for the NMR-based metabolomic analysis; 1 mM TSP was used as the internal standard in the prepared samples^31^.

### Nuclear magnetic resonance (NMR) spectroscopy

A DD2 Nuclear Magnetic Resonance (NMR) spectrometer (Agilent, USA) was utilized for the 1H NMR metabolomics assay. High-resolution NMR spectra were acquired at 298 K using the Agilent DD2 spectrometer at 600 MHz for 1H, which was equipped with an actively shielded gradient unit with a maximum gradient strength output of 65 G cm^−1^. Standard 1D 1H spectra were acquired using the pulse sequence, –RD–90°–t1–90°–tm–90°–acquire–, with a relaxation delay (RD) of 2 s, a mixing time (tm) of 100 ms and a fixed t1 delay of 3 ms. Water suppression was achieved by pre-saturating the water signal during the RD and mixing time. Each spectrum consisted of 256 free induction decays (FIDs) collected into 32 K complex data points with a spectral width of 9,615.38 Hz and an acquisition time of 4.0 s. The metabolite concentrations were assessed for 5 samples by integrating selected NMR resonances from the standard 1-dimensional spectra relative to that of TSP after polynomial baseline correction using the same RD and acquisition parameters listed above. All the samples were equally analyzed in the mode of 64 scans per t1 increment; basically, it took 7.5 min to acquire the 1H NMR spectra of the metabolites produced by each sample.

### Chemometric analysis

The raw 1H NMR spectra were transformed into a three-dimensional data matrix using the MestReNova.9.0.1 software. Briefly, the raw 1H NMR spectra were firstly subjected to the suppression of H_2_O signal and further aligned for the shifted baseline and drifted chemical shift. Then the spectra with chemical shift (<0.00 ppm and >10.00 ppm) were removed completely before the left was binned with 0.03 ppm and transformed into three-dimensional data matrix (Sample ID-Chemical Shift-Signal Abundance). To characterize and identify the differentiable metabolite features, the metabolometric analysis of the data matrix was performed using Metaboanalyst version 3.0 and supervised partial least-squares discriminate analysis (PLS-DA)^27^,^28^,^29^,^32^,^33^,^34^. To reduce the influences of non-biological factors (noise) during pattern recognition analysis, data matrixes of The metabolites (spectral variables) were first normalized to the actual volume of urine for *E. coli* culture, further normalized to CFU/ml (2/50) and the sum of all signals, because those factors all yielded substantial influence on group classification caused really by biological factors such as different *E. coli* strains, and the sort and number of siderophores, finally the distribution was auto-scaled to ensure equal contributions from each metabolic variable to the models. A heatmap was employed to visually display the distribution of all the data and to characterize the relative levels of the metabolites throughout all sample groups. All the different metabolites were recognized and characterized using the loading plots and VIP value (cut-off >1) from the PLS-DA analysis; then they were provisionally identified by combining the information from a local database; open-source databases, including the Human Metabolome Database (HMDB) (http://www.hmdb.ca)^35^ and the Madison Metabolomics Consortium Database (MMCD) (http://mmcd.nmrfam.wisc.edu)^36^; and selected publications^37^,^38^,^39^,^40^,^41^,^42^,^43^. The metabolic pathways hosting the different metabolites were constructed by cross referencing the KEGG database (http://www.kegg.jp)^44^ and the Metaboanalyst 3.0 database (http://www.metaboanalyst.ca/MetaboAnalyst)^32^,^33^,^34^.

### Statistical analysis

The statistics and graphs were generated using Microsoft Office Excel.

## Results

### Global metabolic profiling interrogates the individualized interactive metabolome of UPEC and human urine, which was significantly different from that of non-UPEC and human urine

**Figure 2:**
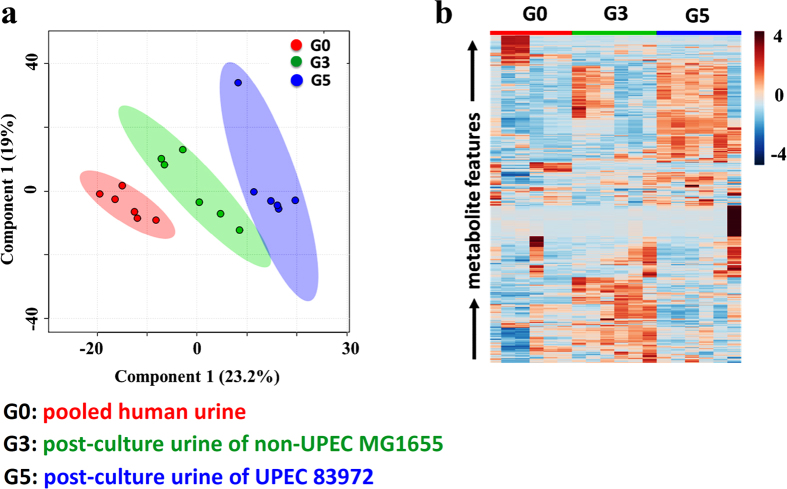
The individualized interactive metabolome between UPEC and human urine was phenotyped and has the capacity to significantly distinguish UPEC from non-UPEC, as UPEC exerted a much stronger influence on the interactive metabolome in cultured human urine than non-UPEC. (**a**) Score plot resulting from the PLS-DA analysis of the interactive metabolomes between the human urine that had been cultured with UPEC or non-UPEC and the pre-culture pooled human urine. (**b**) Heatmap visualizing the relative levels of the interactive metabolomes between the human urine that had been cultured with UPEC or non-UPEC and the pre-culture pooled human urine.

To determine whether the interactive metabolome between the pathogen and human urine has the capacity to differentiate UPEC and non-UPEC, an NMR-based metabolomic method was explored and exploited to provide a global metabolic profile of the interactive metabolome and carry out relevant comparisons to the pre-culture pooled human urine. Representative urinary metabolic profiles for the defined strains and corresponding pre-culture human urine samples are shown in Figure S1. Our results demonstrated that an interactive metabolome was indeed present between the UPEC and cultured human urine (Figure S2) and was significantly different from that between non-UPEC and cultured human urine. The former metabolome incurred much stronger phenotypic perturbations in the interactive metabolome (Fig. 2a). Consistent with a previous targeted assay of the interactive metabolome between UPEC and cultured human urine (data not shown), the utilized metabolome (metabolic cost) and excreted metabolome (metabolic donation) present in the cultured human urine enabled us to significantly distinguish the UPEC from non-UPEC, as well as the pre-culture human urine (Fig. 2b).

### Siderophore biosynthesis markedly regulated the interactive metabolome of UPEC and human urine and was dependent on the number and types of siderophores

**Figure 3:**
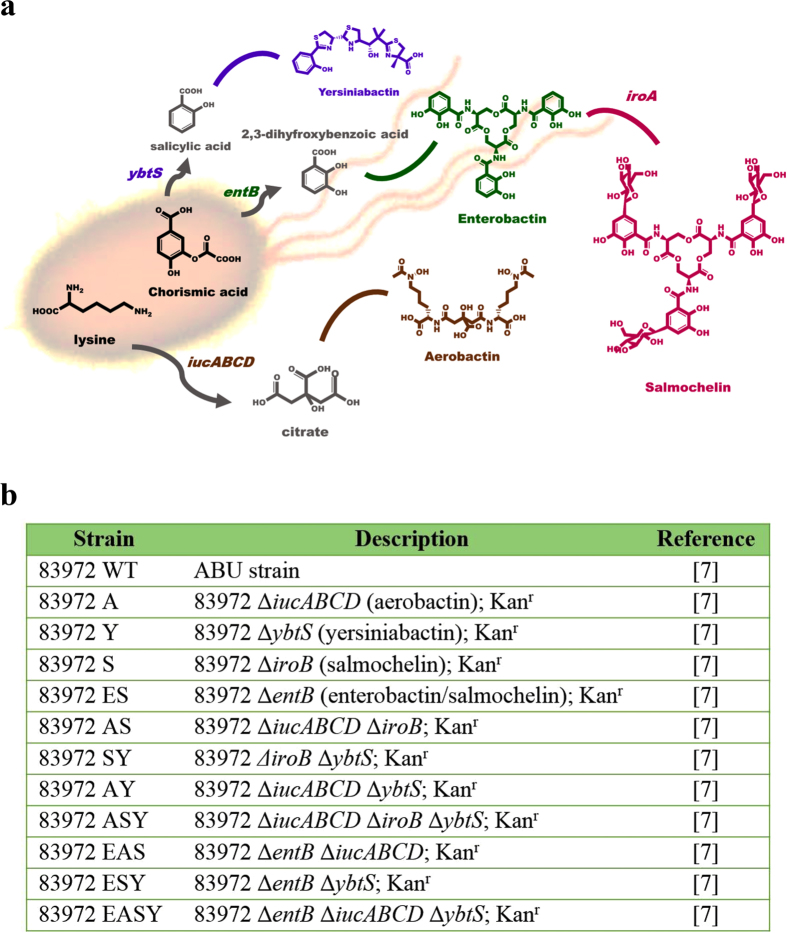
A genetic strategy was employed to prepare the UPEC mutant strains with single, double, triple and quadruple deletions of siderophores. (**a**) Schematic illustration of the biosynthetic pathways for the siderophores aerobactin, salmochelin, enterobactin and yersiniabactin. (**b**) The selected UPEC and mutant strains used in this study.

**Figure 4:**
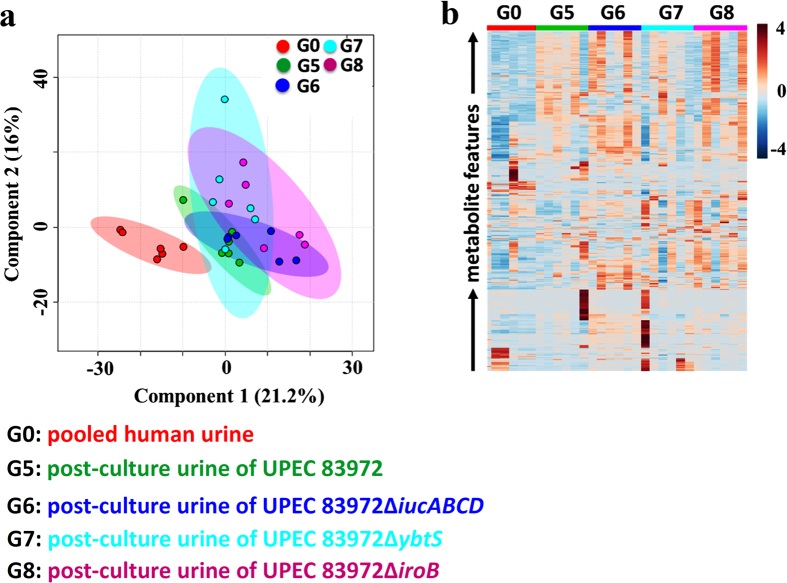
The deletion of a single siderophore has almost no impact on the interactive metabolome between UPEC and human urine. (**a**) Score plot resulting from the PLS-DA analysis of the interactive metabolomes between the human urine that had been cultured with the selected strains and the pre-culture pooled human urine. (**b**) Heatmap visualizing the relative levels of the interactive metabolomes between the human urine that had been cultured with the selected strains and pre-culture pooled human urine.

**Figure 5:**
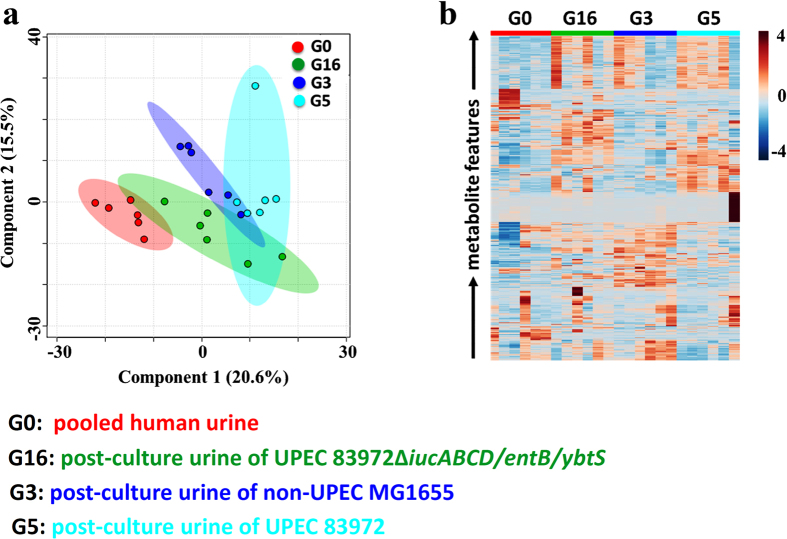
Siderophores coordinately modulated the virulence-associated interactive metabolome, as the complete deletion of all four siderophores restored the perturbations in the interactive metabolome to the levels observed in the interactive metabolomes with non-UPEC and human urine. (**a**) Score plot resulting from the PLS-DA analysis of the interactive metabolomes between the human urine that had been cultured with the selected strains and the pre-culture pooled human urine. (**b**). Heatmap visualizing the relative levels of the interactive metabolomes between the human urine that had been cultured with the selected strains and the pre-culture pooled human urine.

To investigate the regulatory effects of siderophore biosynthesis on the interactive metabolome between UPEC and human urine, we selected UPEC and its associated mutant strains with different patterns of deletion of four siderophores (Fig. 3) to facilitate the interactive metabolome assay compared to pre-cultured human urine. Typical urinary metabolic profiles for the defined UPEC and mutant strains are shown in Figure S3. The global metabolomics assay revealed that the deletion of a single siderophore had almost no impact on the UPEC-induced perturbations of the interactive metabolome in human urine (Fig. 4). Furthermore, the simultaneous deletion of two siderophores fully restored the perturbed interactive metabolome to the control level in pre-cultured human urine, suggesting that those siderophores jointly regulate the perturbed interactive metabolome in cultured human urine by interacting with UPEC (Figure S4). As the number of deleted siderophores increased, the impact of UPEC on the perturbations in the interactive metabolome in cultured human urine decreased markedly (Figure S5), and the impacts of salmochelin and aerobactin deletions on the interactive metabolome were much stronger than those of the enterobactin and yersiniabactin deletions. Surprisingly, the loss of all four siderophores exerted the strongest regulatory effects upon the UPEC-induced perturbations in the interactive metabolome in cultured human urine (Fig. 5). Based on these results, we can argue that the siderophores coordinately regulated the interactive metabolome between UPEC and human urine, rather than any single siderophore.

### Siderophores coordinately modulated the virulence-associated interactive metabolome between UPEC and human urine

**Figure 6:**
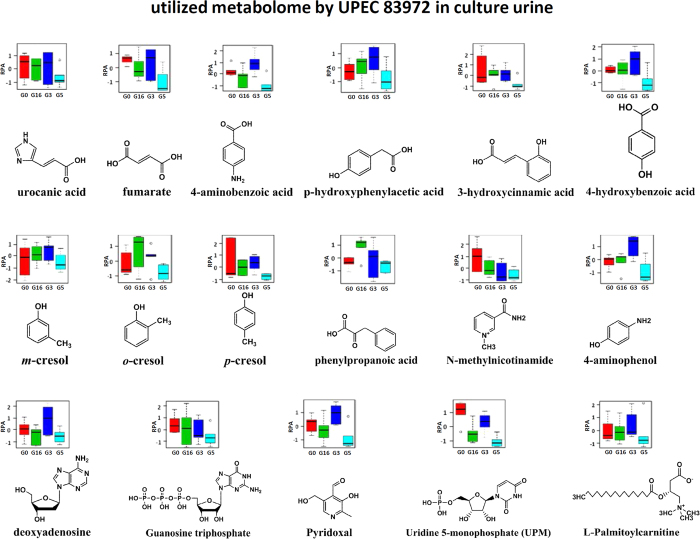
The different metabolites hosted by the identified utilized metabolome were characterized according to their chemical structures and relative levels (RPA) in the human urine that had been cultured with UPEC (G5), non-UPEC (G3), and UPEC with the deletion of all four siderophores (G16), as well as the pre-culture pooled human urine (G0).

**Figure 7:**
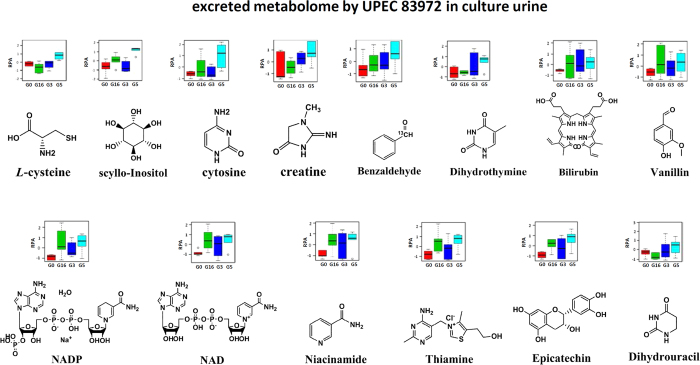
The different metabolites hosted by the identified excreted metabolome of the interactive metabolome were characterized according to their chemical structures and relative levels (RPA) in the human urine that had been cultured with UPEC (G5), non-UPEC (G3), and UPEC with the deletion of all four siderophores (G16), as well as the pre-culture pooled human urine (G0).

**Figure 8:**
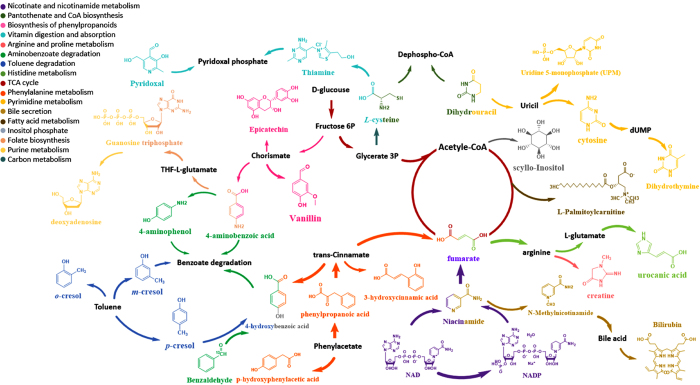
The metabolic pathways hosted in the identified interactive metabolome included 17 metabolites in human urine (utilized metabolome) that were significantly decreased by the UPEC treatment and 14 metabolites in human urine (Excreted metabolome) that were markedly enhanced by the UPEC treatment. Of the 31 different metabolites, 17 primarily affected metabolic pathways, including those involving amino acid metabolism, energetic metabolism, gut microbe metabolism, fatty acid metabolism, purine metabolism, and carbon metabolism.

To determine the relationship between the interactive metabolome modulated by siderophore biosynthesis and the virulence of UPEC, we compared the interactive metabolomes present in the urine that had been cultured with UPEC, non-UPEC and UPEC lacking all siderophores, as well as the pre-culture human urine. Our data clearly demonstrated that the complete loss of siderophores was capable of substantially restoring the interactive metabolome to the control level in the pre-cultured human urine, and the metabolic cluster of the interactive metabolome in the score plot almost completely overlapped with the interactive metabolome influenced by non-UPEC and is closer to the pre-cultured urine than the non-UPEC (Fig. 5). This result might suggest that the characterized interactive metabolome was mechanistically associated with the virulence of UPEC, as siderophore biosynthesis enabled us to distinguish UPEC from non-UPEC by modulating the virulence-associated interactive metabolome. Aerobactin and salmochelin demonstrated much stronger modulatory effects on the interactive metabolome, and they might be potential targets for further chemical interventions against UPEC-induced UTI. Finally, the virulence-associated interactive metabolome was provisionally identified as the utilized metabolome and excreted metabolome; the former primarily accounts for the metabolic cost when UPEC was incubated in the pooled human urine, and the latter was attributed to the metabolic donations from UPEC to the cultured human urine in the form of chemicals. The identified interactive metabolomes correlated closely with the virulence of UPEC, as they contained 31 metabolites, of which 17 were identified as part of the utilized metabolome (urocanic acid, fumarate, p-aminobenzoic acid, p-hydroxyphenylacetic acid, uridine 5-monophosphate (UPM), 4-hydroxybenzoic acid, 3-hydroxycinnamic acid, trans-2-hydroxycinnamate, m-cresol, o-cresol, p-cresol, 4-aminophenol, N-methylnicotinamide, phenylpyruvic acid, deoxyadenosine, guanosine triphosphate, pyridoxal and L-palmitoylcarnitine) and were decreased significantly by UPEC (Fig. 6), whereas the others were considerably enhanced and were identified as part of the excreted metabolome (L-cysteine, scyllo-inositol, cytosine, creatinine, vanillin, bilirubin, dihydrothymine, benzaldehyde, NADP, NAD, niacinamide, thiamine, epicatechin and dihydrouracil) (Fig. 7). Sixteen metabolic pathways were annotated to host those metabolites involved in energetic metabolism, amino acid metabolism, gut microbe metabolism, and other processes (Fig. 8). The changes in these activities mechanistically highlighted the capability of siderophore biosynthesis for differentiating UPEC from non-UPEC.

## Discussion

Human urine is the fundamental medium for UPEC-induced infection because the strains acquire the essential carbohydrate sources from urine to facilitate normal growth *in vivo* that leads to the development of UTI. Moreover, the composition of human urine has a noticeable influence on the virulence of UPEC^10^,^11^. Generally, chemical exchange primarily accounts for metabolic interaction of UPEC and human urine and can be characterized as the “interactive metabolome”, which includes the utilized metabolome (metabolic cost) and excreted metabolome (metabolic donation) triggered by the interaction between UPEC and cultured human urine, by which the strains are capable of maintaining pathogenic growth in women’s bladders as the fundamental driver for causing infection^45^,^46^,^47^,^48^,^49^. In this study, we employed a combination of metabolomics and genetics^4^,^5^,^6^ to discover and characterize the individualized interactive metabolome between UPEC and human urine that should be significantly differentiated from that between non-UPEC and human urine when the defined strains are cultured in pooled human urine. As expected, UPEC exerted a much stronger influence upon the interactive metabolome present in cultured human urine than non-UPEC, and siderophores coordinately modulated the interactive metabolome implicated in the virulence of UPEC, while the complete deletion of the siderophores in the UPEC strain was found to overlap with non-UPEC in modifying the interactive metabolome in cultured human urine. These results suggest that the absence or presence of siderophores is the key determinant for differentiating UPEC from non-UPEC and is mostly consistent with our previous studies, as siderophore biosynthesis was essential for enhancing UPEC virulence^2^,^5^,^6^. Basically, enterobactin is one conserved siderophore produced by both uropathogenic and pathogenic *E. coli*, the fact convinced us that enterobactin is not directly linked to the virulence of pathogens although it is the key precursor of salmochelin biosynthesis. Consequently, we deduced that aerobactin and salmochelin clearly had much stronger modulatory effects upon the virulence-associated interactive metabolome compared to enterobactin and yersiniabactin, even though the combination of these two siderophores rendered the strongest impact. Importantly, this study reports, for the first time, that aerobactin is observably linked to UPEC virulence by substantially modulating the individualized interactive metabolome in coordination with other siderophores.

The identified interactive metabolome between UPEC and human urine participated primarily in 16 central metabolic pathways (Fig. 8), including energetic metabolism, amino acid metabolism, carbon metabolism, gut microbe metabolism, and purine metabolism. This discovery was consistent with a previous study^50^, as peptides and amino acids are the primary carbon source for UPEC during infection of the urinary tract, and central metabolic pathways are necessary for UPEC fitness *in vivo*. Moreover, these pathways could be considered critical components of virulence for pathogenic microbes^2^,^3^,^6^,^51^,^52^,^53^.

## Conclusion

In the present study, we discovered and characterized the first individualized interactive metabolome between UPEC and human urine by employing a combination of metabolomics and genetics, and we showed that the interactive metabolome is involved in modulating the virulence of UPEC during infection. In addition, we confirmed that siderophore biosynthesis coordinately modulates the virulence-associated interactive metabolome and can significantly differentiate UPEC from non-UPEC. This study provides novel insights into the virulence of UPEC and associated UTI pathogenesis and might lead to development of virulence-based therapeutic interventions and the discovery of drugs to treat pathogenic infections.

## Additional Information

**How to cite this article**: Su, Q. *et al*. Siderophore biosynthesis coordinately modulated the virulence-associated interactive metabolome of uropathogenic *Escherichia coli* and human urine. *Sci. Rep*. **6**, 24099; doi: 10.1038/srep24099 (2016).

## Supplementary Material

Supplementary Information

## Figures and Tables

**Figure 1:**
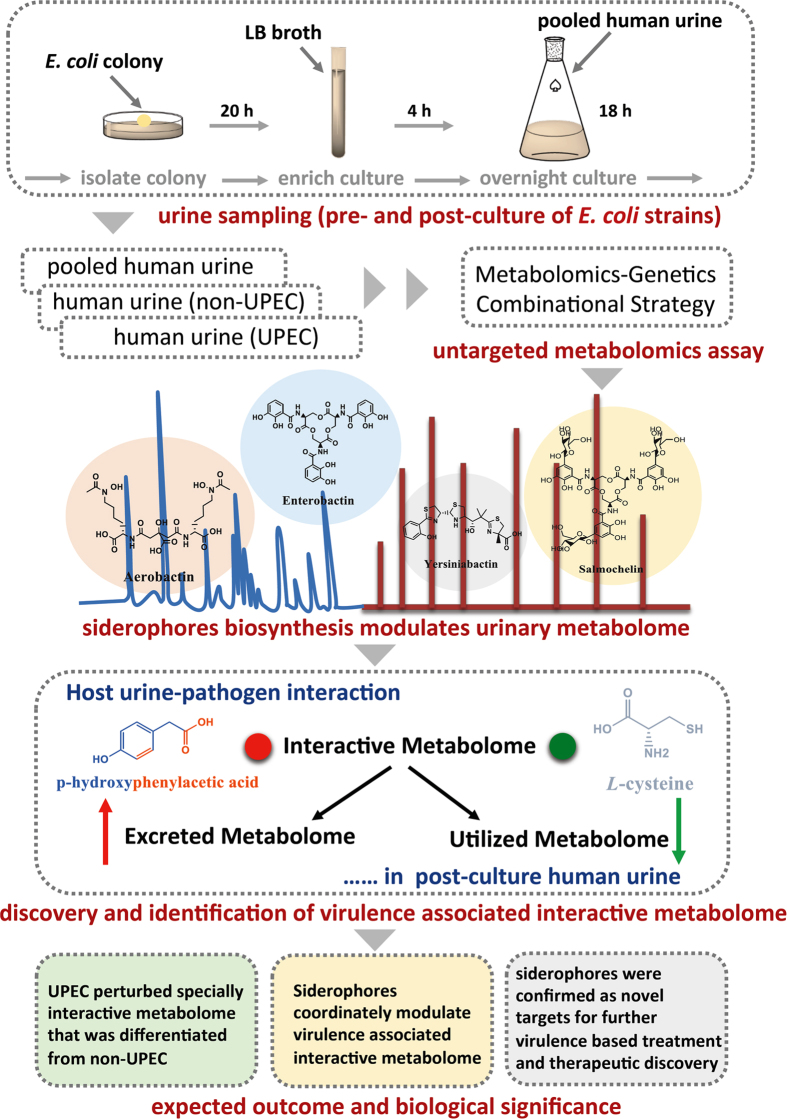
Schematic illustration of the strategy utilized in this study. Briefly, the sampling procedure was manipulated to incubate all the defined strains with the pooled human urine samples, and then an NMR-based metabolomics analysis was performed to discover the individualized interactive metabolome (utilized metabolome and excreted metabolome) between UPEC and human urine that can differentiate UPEC from non-UPEC. A combination of metabolomics and genetics was employed to elucidate how the siderophores coordinately modulated the virulence-associated interactive metabolome. Finally, our data provide novel insights into the UPEC-human urine interaction and show that siderophore biosynthesis has substantial modulatory effects on the interactive metabolome as the critical components of UPEC virulence. Siderophores may be novel targets for further chemical interventions and the discovery of drugs to treat UPEC-induced urinary tract infection.
