# Supplementary material for: Siderophore biosynthesis coordinately modulated the virulence-associated interactive metabolome of uropathogenic Escherichia coli and human urine
Source: Sci Rep. 2016 Apr 14;6:24099. doi: 10.1038/srep24099 (PMC4831015; doi:10.1038/srep24099)
Supplement: Supplementary Information [file srep24099-s1.pdf]

Supplementary Tables 1-3 and Figures 1-5

Siderophore biosynthesis coordinately modulated the virulence-associated interactive metabolome of uropathogenic *Escherichia coli* and human urine

Qiao Su[1], Tianbing Guan[1] and Haitao Lv[1, 2]*

[1]The Laboratory for Functional Omics and Innovative Chinese Medicine, Innovative Drug Research Center, Chongqing University, Chongqing 401331, *P.R.* China

[2]Shanghai Center for Systems Biomedicine, Key Laboratory of Systems Biomedicine (Ministry of Education), Shanghai Jiao Tong University, Shanghai 200240, China

***Corresponding author.**

Haitao Lv Ph.D., Professor. Tel: +86-23-65678464; Fax: +86-23-65678450; Email.: haitao.lu@cqu.edu.cn

Table S1. Physiological information for the healthy volunteers

Urine Type	Morning Urine
Age	22-25
Male	22
Female	15

Table S2. The selected UPEC and associated mutant strains (References 4-6, 20 were listed in text).

Strain	Description	Reference or
**Urine**	Fresh Urine	This study
**MG1655**	MG1655	[4-6]
**83972 WT**	ABU strain	[20]
**83972 A**	83972 *ΔiucABCD* (aerobactin); Kan^r	[20]
**83972 Y**	83972 *ΔybtS* (yersiniabactin); Kan^r	[20]
**83972 S**	83972 *ΔiroB* (salmochelin); Kan^r	[20]
**83972 ES**	83972 *ΔentB* (enterobactin/salmochelin);	[20]
**83972 AS**	83972 *ΔiucABCD ΔiroB;* Kan^r	[20]
**83972 SY**	83972 *ΔiroB ΔybtS;* Kan^r	[20]
**83972 AY**	83972 *ΔiucABCD ΔybtS;* Kan^r	[20]
**83972**	83972 *ΔiucABCD ΔiroB ΔybtS;* Kan^r	[20]
**83972**	83972 *ΔentB ΔiucABCD;* Kan^r	[20]
**83972**	83972 *ΔentB ΔybtS;* Kan^r	[20]
**83972**	83972 *ΔentB ΔiucABCD ΔybtS;* Kan^r	[20]

Table S3: Primers used in this study for the deletion of single, double, triple and quadruple siderophores from UPEC strain involving aerobactin, salmochelin, yerisniabactin and enterobactin.

Primer	Sequence (5'-3')
***iucABCD* deletion F primer**	GAGCTGTTTGACTATGACCCTGCCCTCTGAAAAACCAGCCACAGATGTGGGTGTAGGCTGGAGCTGCTTC
***iucABCD* deletion R primer**	GCGGGCGGCATACTGAGATCGAATAAATCACGCCCCAGTACGCGATTAAGCATATGAATATCCTCCTTAG
***iucABCD* screening F primer**	TTGATAATGAGAATCATTATTGAC
***iucABCD* screening R primer**	GTGGTTTGCGCCATCTCCGC
***ybtS* deletion F primer**	AGGTTAGAAAACAGTTACTCCTACACCATTAAATAGGGCGCAATGCTCGCGTGTAGGCTGGAGCTGCTTC
***ybtS* deletion R primer**	CCGGCTGCATTCGGCAAGAGAACTGATGACAAGCGCAATTGATTGATCCGCATATGAATATCCTCCTTAG
***ybtS* screening F primer**	GGCAAATTACCACCACCTCC
***ybtS* screening R primer**	GTTATCGGTGGCGTTGTTG
***entB* deletion F primer**	CGGTCGGGAAAGTCGATAAAAAACAATTACGTCAGTGGCTGGCGTCACGCGTGTAGGCTGGAGCTGCTTC
***entB* deletion R primer**	TGAAATCCATTATTTCACCTCGCGGGAGAGTAGCTTCCACCAGGCGTCGACATATGAATATCCTCCTTAG
***entB* screening F primer**	CATGGAAGATGAGCTGATGG
***entB* screening R primer**	GTCGCTCCGTTTCAGCTAAC
***iroB* deletion F primer**	TCTGTAAAATACGATCCACTGGCCGGATCGTTCCGCAAAAAAGCCAGCACGTGTAGGCTGGAGCTGCTTC
***iroB* deletion R primer**	TGCGTCGACTGCCTGATTTAGATCGTCAAGCGGAGAGGGATTTTCTCATGCATATGAATATCCTCCTTAG
***iroB* screening F primer**	TCACAGCCTGTCTTGTCAGC
***iroB* screening R primer**	TGTCCATAACGCTGGTGAAG

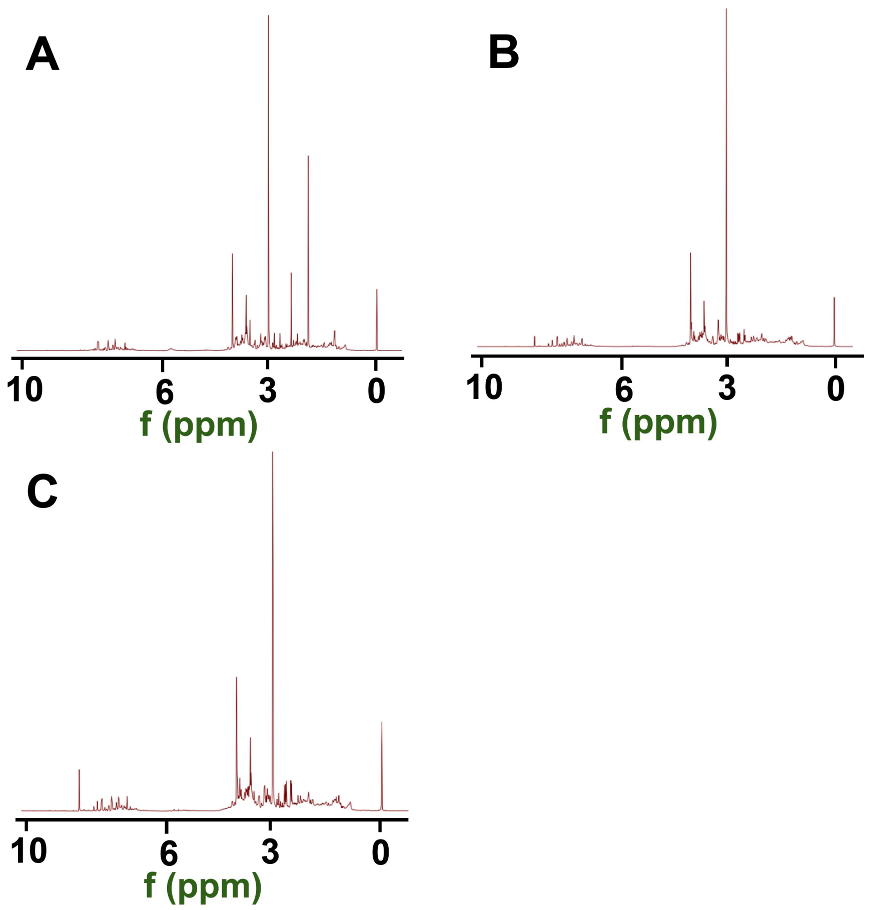

Figure S1. Typical NMR based urinary metabolic profiles of (A) pre-culture pooled human urine; (B) post-culture pooled human urine of non-UPEC strain; (C) post-culture pooled human urine of UPEC strain.

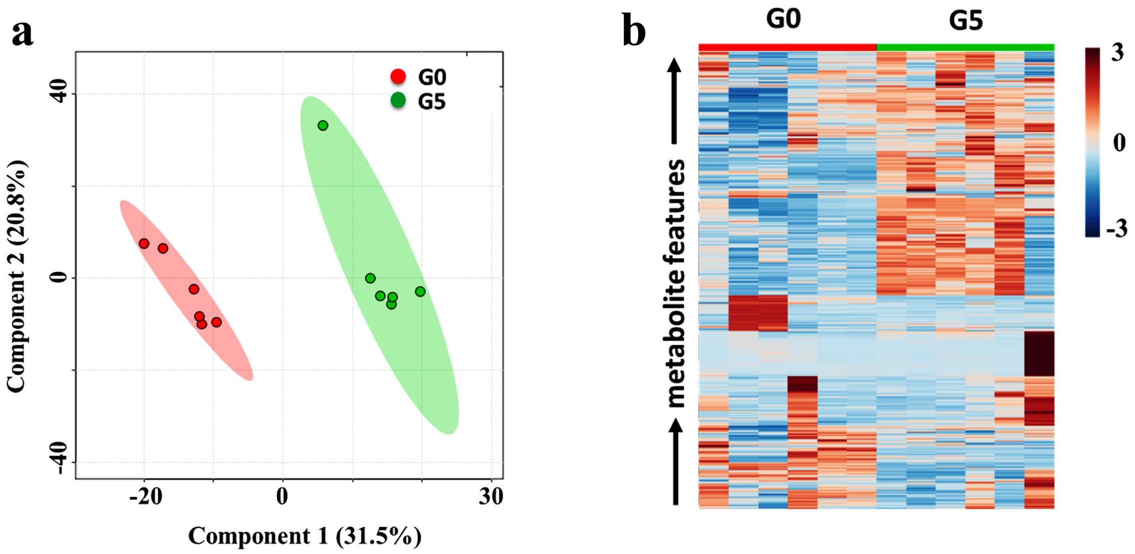

G0: pooled human urine

G5: post-culture urine of UPEC 83972

Figure S2. The differentiable interactive metabolome was visualized and characterized by the shifted metabolic clusters between post-culture pooled human urine of UPEC strain and pre-culture pooled human urine. A) Score plot resulted from PLS-DA analysis of interactive metabolome amongst the post-culture pooled human urine of UPEC and pre-culture pooled human urine. B) heatmap visualized the relative level of interactive metabolome amongst the post-culture pooled human urine of UPEC and pre-culture pooled human urine.

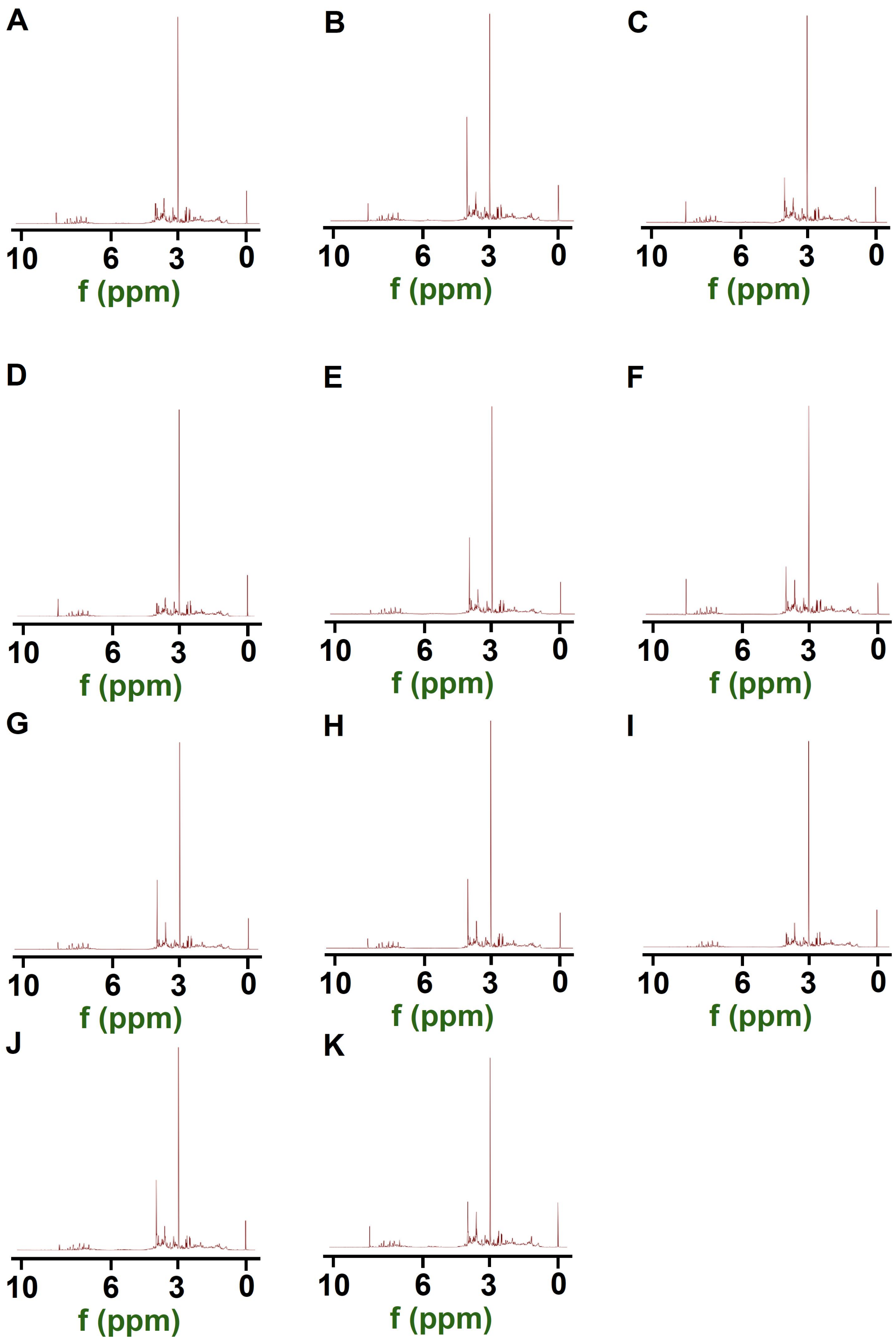

Figure S3. Typical NMR based urinary metabolic profiles of (A) post-culture pooled human urine of 83972*ΔiucABCD*; (B) post-culture pooled human urine of 83972 *ΔybtS* (yersiniabactin); (C) post-culture pooled human urine of 83972 *ΔiroB* (salmochelin); (D) post-culture pooled human urine of 83972 *ΔentB*

(enterobactin/salmochelin); (E) post-culture pooled huamn urine of 83972 *ΔiucABCD ΔiroB*; (F) post-culture pooled human urine of 83972 *ΔiroB ΔybtS*; (G) post-culture pooled human urine of 83972 *ΔiucABCD ΔybtS*; (H) post-culture pooled human urine of 83972 ΔiucABCD *ΔiroB ΔybtS*; (H) post-culture pooled human urine of 83972 ΔiucABCD *ΔiroB ΔybtS*; (I) 83972 *ΔentB ΔiucABCD* (J) post-culture pooled human urine of 83972 *ΔentB ΔybtS*; (K) post-culture pooled human urine of 83972 *ΔentB ΔiucABCD ΔybtS.*

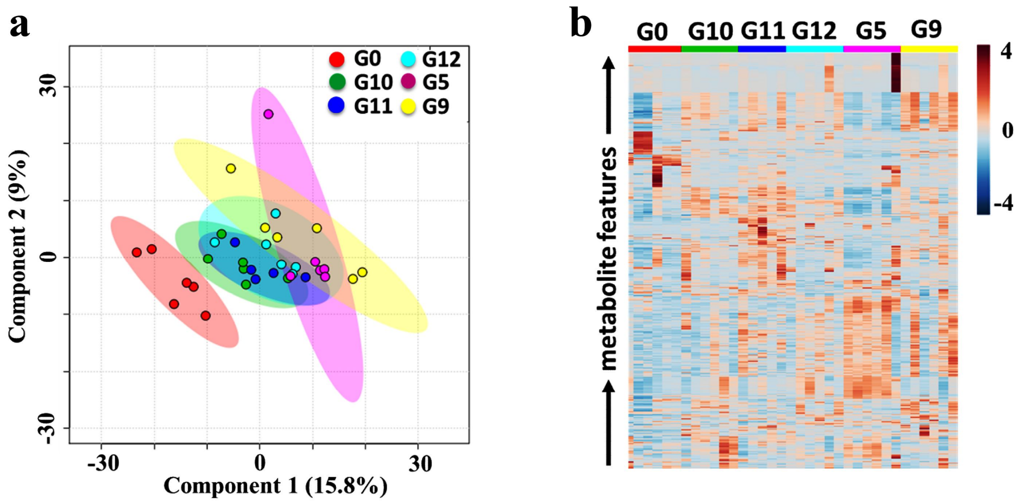

G0: pooled human urine

G10: post-culture urine of UPEC 83972Δ*iucABCD/iroB*

G11: post-culture urine of UPEC 83972Δ*iroB/ybtS*

G12: post-culture urine of UPEC 83972Δ*iucABCD/ybtS*

G5: post-culture urine of UPEC 83972

G9: post-culture urine of UPEC 83972Δ*entB*

Figure S4. The shutdown of double siderophores rendered a certain influence on the interactive metabolome between UPEC and human urine. A. Score plot resulted from PLS-DA analysis of interactive metabolome amongst post-culture human urine of the selected strains and pre-culture pooled human urine. B. Heatmap visualized the relative level of interactive metabolome amongst post-culture human urine of the selected strains and pre-culture pooled human urine.

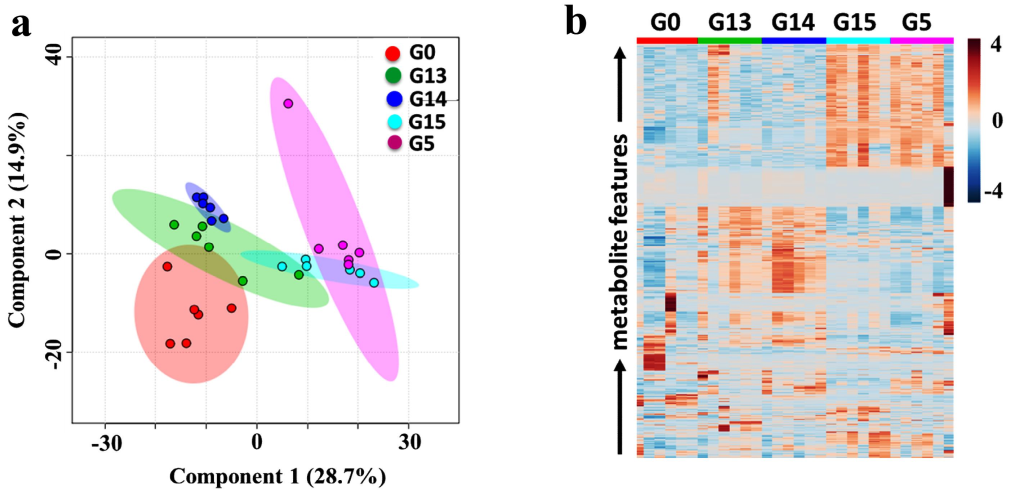

G0: pooled human urine
G13: post-culture urine of UPEC 83972Δ*iucABCD/iroB/ybtS*
G14: post-culture urine of UPEC 83972Δ*entB/iucABCD*
G15: post-culture urine of UPEC 83972Δ*entB/ybtS*
G5: post-culture urine of UPEC 83972

Figure S5. The deletion of triple siderophores exerted significantly influence on the interactive metabolome between UPEC and human urine with the tendency statistically restored to control level in pre-culture pooled human urine. A. Score plot resulted from PLS-DA analysis of interactive metabolome amongst post-culture human urine of the selected strains and pre-culture pooled human urine. B. Heatmap visualized the relative level of interactive metabolome amongst post-culture human urine of the selected strains and pre-culture pooled human urine.
